# Reversible manifestations of extraparenchymal neurocysticercosis

**DOI:** 10.1002/ccr3.1602

**Published:** 2018-05-22

**Authors:** Edison M. Campos, Flavius D. Raslau, Robert Salinas, Daniela Di Capua, John T. Slevin, Mauricio F. Villamar

**Affiliations:** ^1^ Department of Neurology Hospital de Especialidades Eugenio Espejo Quito Ecuador; ^2^ Department of Radiology University of Kentucky Lexington KY USA; ^3^ Department of Neurology University of Kentucky Lexington KY USA

**Keywords:** hydrocephalus, movement disorders, neurocysticercosis, neuroinfectious disease, *Taenia solium*

## Abstract

Movement disorders are uncommon manifestations of neurocysticercosis. When present, most are secondary to parenchymal lesions in the basal ganglia. Rarely, movement disorders can occur in racemose/extraparenchymal neurocysticercosis, an aggressive variant frequently associated with cerebrospinal fluid outflow obstruction and hydrocephalus. Appropriate treatment can reverse neurological manifestations.

A 21‐year‐old Ecuadorian woman with previous hydrocephalus due to neurocysticercosis and ventriculoperitoneal shunt placement at age 19 presented with ophthalmoparesis, cerebellar outflow tremor, and bilaterally upgoing toes (Video S1, pretreatment). CSF opening pressure was 16 cm H_2_O. Figure 1A shows MRI.

**Figure 1:**
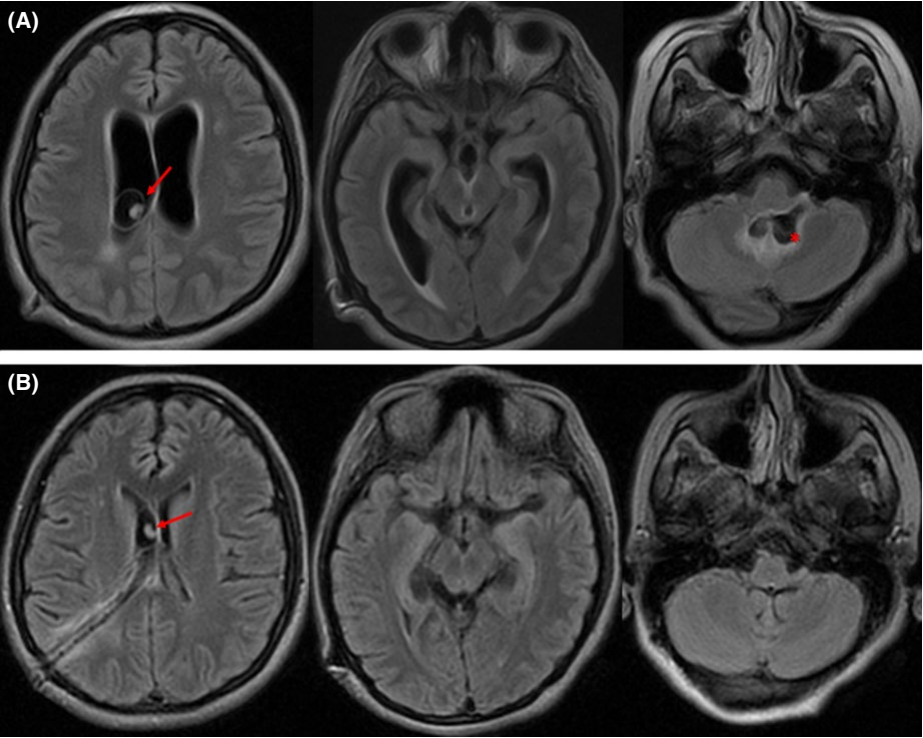
MRI FLAIR sequence. A, Pretreatment images demonstrate communicating hydrocephalus with transependymal CSF egress. Note the intraventricular cyst with scolex (arrow) in the right lateral ventricle, and another cyst in the fourth ventricle (asterisk). B, Post‐treatment images, obtained 3 weeks later, show resolution of hydrocephalus and fourth ventricle cyst

After 3 weeks of treatment with steroids and albendazole, there was clinical and radiological improvement (Video S1, post‐treatment; Figure 1B).

Movement disorders are a rare manifestation of neurocysticercosis. Basal ganglia involvement can cause chorea and/or dystonia. Racemose/extraparenchymal neurocysticercosis, an aggressive variant that commonly causes CSF outflow obstruction and hydrocephalus, can present with parkinsonism, cerebellar outflow tremor, cranial neuropathies, and/or corticospinal signs.^1^, ^2^

## CONFLICT OF INTEREST

None declared.

## AUTHOR CONTRIBUTIONS

EMC: involved in the case concept and design, acquisition of data, interpretation of data, and manuscript writing. FDR: performed neurodiagnostic evaluation and critically revised the manuscript for intellectual content. RS: involved in the case concept and design, acquisition of data, interpretation of data, and critically revised the manuscript for intellectual content. DDC: involved in the case concept and design, acquisition of data, interpretation of data, and critically revised of the manuscript for intellectual content. JTS: interpreted the data and critically revised the manuscript for intellectual content. MFV: involved in the case concept and design, interpretation of data, manuscript writing, and critically revised the manuscript for intellectual content.
